# The 3' untranslated region of human *Cyclin-Dependent Kinase 5 Regulatory subunit 1 *contains regulatory elements affecting transcript stability

**DOI:** 10.1186/1471-2199-8-111

**Published:** 2007-12-03

**Authors:** Silvia Moncini, Annamaria Bevilacqua, Marco Venturin, Claudia Fallini, Antonia Ratti, Angelo Nicolin, Paola Riva

**Affiliations:** 1 Department of Biology and Genetics, Medical Faculty, University of Milan, Via Viotti 3/5, 20133 Milan, Italy; 2 Department of Pharmacology, Chemotherapy and Medical Toxicology, University of Milan, Via Vanvitelli 32, 20129 Milan, Italy; 3 Department of Neuroscience, University of Milan, IRCCS Istituto Auxologico Italiano, Via Zucchi 18, 20095 Cusano, Milan, Italy

## Abstract

**Background:**

*CDK5R1 *plays a central role in neuronal migration and differentiation during central nervous system development. *CDK5R1 *has been implicated in neurodegenerative disorders and proposed as a candidate gene for mental retardation. The remarkable size of *CDK5R1 *3'-untranslated region (3'-UTR) suggests a role in post-transcriptional regulation of *CDK5R1 *expression.

**Results:**

The bioinformatic study shows a high conservation degree in mammals and predicts several AU-Rich Elements (AREs). The insertion of *CDK5R1 *3'-UTR into luciferase 3'-UTR causes a decreased luciferase activity in four transfected cell lines. We identified 3'-UTR subregions which tend to reduce the reporter gene expression, sometimes in a cell line-dependent manner. In most cases the quantitative analysis of luciferase mRNA suggests that CDK5R1 3'-UTR affects mRNA stability. A region, leading to a very strong mRNA destabilization, showed a significantly low half-life, indicating an accelerated mRNA degradation. The 3' end of the transcript, containing a class I ARE, specifically displays a stabilizing effect in neuroblastoma cell lines. We also observed the interaction of the stabilizing neuronal RNA-binding proteins ELAV with the CDK5R1 transcript in SH-SY5Y cells and identified three 3'-UTR sub-regions showing affinity for ELAV proteins.

**Conclusion:**

Our findings evince the presence of both destabilizing and stabilizing regulatory elements in *CDK5R1 *3'-UTR and support the hypothesis that *CDK5R1 *gene expression is post-transcriptionally controlled in neurons by ELAV-mediated mechanisms. This is the first evidence of the involvement of 3'-UTR in the modulation of *CDK5R1 *expression. The fine tuning of *CDK5R1 *expression by 3'-UTR may have a role in central nervous system development and functioning, with potential implications in neurodegenerative and cognitive disorders.

## Background

*CDK5R1 *(*Cyclin-dependent kinase 5 regulatory subunit 1*) encodes for p35, a protein required for the activation of cyclin-dependent kinase 5 (CDK5), whose activity plays a key role in central nervous system development [1]. Monomeric CDK5 does not show any enzymatic activity, requiring association with its regulatory partners p35 or p39. During neuronal migration, p35-activated CDK5 phosphorylates NudEL, the homologous of an Aspergillus nidulans gene involved in nuclear translocation and in cytoskeletal organization of migrating neurons by dynein regulation [2]. In mouse, Cdk5 modulates PAK kinases and is implicated in actin reorganization, which may be critical for neuron migration along radial glia [3]. In mice lacking Cdk5 or p35, abnormal formation of cortical layers occurs: a loss of the Cdk5 pathway appears to affect later migrating neurons as the cortical layers emerge from the cortical plate [4,5], leading to severe cortical lamination defects, adult mortality and seizures [5,6]. The absence of p39 did not produce aberrant phenotype, indicating a predominant role of p35 in CDK5 activation [7]. It is also known that both Cdk5 and p35 concentrate at the leading edges of axonal growth cones and have been shown to regulate neuritis outgrowth in cortical neuron culture [8]. The active CDK5/p35 complex is involved in further processes required for central nervous system development and function, such as axonal regeneration [9], cellular differentiation, neuronal apoptosis [10], learning and memory processes [11], synaptic transmission [12] and membrane trafficking during the outgrowth of neuronal processes [13]. Hyperactivity of CDK5 mediated by p25, a proteolytic fragment of p35, has been implicated in the pathogenesis of several neurodegenerative disorders, such as Alzheimer's disease [14], Parkinson's disease [15] and amyotrophic lateral sclerosis [16]. In fact, phosphorylated neurofilaments and their associated kinases, most of which are represented by CDK5, were found in protein aggregates typical of neurodegenerative diseases. Findings on *CDK5R1 *deletion in patients with NF1 microdeletion syndrome showing mental retardation [17,18] and the recently reported *CDK5R1 *mutations in non-syndromic mental retardation patients [19], pinpoint the gene as a candidate for mental retardation susceptibility in NF1 microdeletion syndrome and in a subgroup of non-syndromic mental retarded patients.

More recently the CDK5/p35 complex has also been reported to regulate several activities such as exocytosis, gene transcription, tissue regeneration, senescence, apoptosis and hormone regulation also in extra-neuronal cells [20].

Given the key role of *CDK5R1 *in the development, differentiation and physiology of brain and its involvement in extra-neuronal cell activities, it is conceivable that accurate spatio-temporal regulation of its expression is needed. *CDK5R1 *is characterized by an extended 3'-UTR (2725 bp), which accounts for about 75% of the whole transcript and is among the 5% longest annotated 3'-UTRs [21]. We recently reported the presence of known regulatory elements in *CDK5R1 *3'-UTR such as a potential GY-box motif (GUCUUCC, nt 1341–1347) and three putative AU-Rich Elements (AREs) at the 3' end of the transcript [19]; GY-box has been validated as microRNA target in Drosophila [22,23]; AREs have a well known role in post-transcriptional regulation of mRNA stability and degradation through the binding of specific factors [24]. These features suggest a role for the 3'-UTR in the control of *CDK5R1 *expression.

3'-UTRs have been shown to play crucial roles in a wide variety of regulatory mechanisms [25], including modulation of mRNA stability and degradation [24,26], translation efficiency [27], transport out of the nucleus and sub-cellular localization of mRNA [28]. Expression of several important nervous system genes, including genes encoding neurotransmitter receptors, biosynthetic enzymes, cytoskeletal proteins, growth factors and associated proteins, are known to be regulated by functional elements in their 3'-UTRs [29-33]. Indeed, post-transcriptional regulation exerted by the 3'-UTR has been proposed as an effective counterpart to the mechanism of transcription in fine modulation of gene expression, in particular during the development of the central nervous system [34]. The importance of 3'-UTRs in regulating gene expression is also underlined by the finding that mutations affecting the 3'-UTR can lead to serious pathologies [35,36]. The role of the 3'-UTR for appropriated gene control has been demonstrated [37] and it is possible that certain disorders of neuronal plasticity and learning are due to perturbations in 3'-UTR-mediated functions [35].

Thus, regulatory elements within the 3'-UTR of *CDK5R1 *are likely to be involved in the tuning of its expression during central nervous system development and neuronal migration, acting on mRNA stability, translation efficiency and/or sub-cellular localization. Nevertheless, to our knowledge, studies on *CDK5R1 *3'-UTR function have not yet been reported.

In order to explore the role of *CDK5R1 *3'-UTR we have predicted additional regulatory elements and performed functional studies by means of dual luciferase assays and mRNA quantification, observing that the 3'-UTR decreases the reporter activity in different cell lines and that different regulatory elements are present, some with stabilizing and others with destabilizing function. We have identified regulatory regions showing cell-line specificity, in particular we have identified a region showing a destabilizing effect caused by increased mRNA degradation. We have also observed a stabilizing effect of the 3' end region containing a canonical ARE in two neuroblastoma cell lines and have obtained evidence of binding of the neuronal-specific ELAV (nELAV) proteins to *CDK5R1 *mRNA.

We report the first evidence on the involvement of *CDK5R1 *3'-UTR in the modulation of gene expression. These findings suggest that *CDK5R1 *3'-UTR may contribute to modulate its expression during central nervous system development and functioning. The impairment of this mechanism may be involved in the pathogenesis of both neurodegenerative and cognitive disorders.

## Results

### *CDK5R1 *3'-UTR is highly conserved in mammals and contains several putative regulatory elements

**Figure 1:**
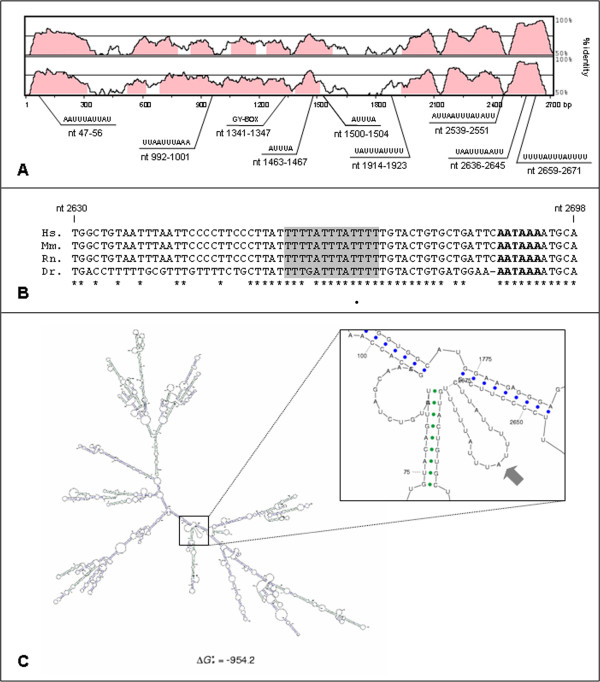
**Bioinformatic analysis of *CDK5R1 *3'-UTR**. A) Human-mouse (above) and human-rat (below) conservation analysis of *CDK5R1 *3'-UTR using the mVISTA tool. Regions of at least 100 bp with identity greater than 70% are indicated in pink. The plots refer to the human *CDK5R1 *3'-UTR sequence (UTRdb ID: 3HSA086450). The position of the predicted regulatory elements is indicated. B) Multiple alignment of the last portion of human (Hs.), mouse (Mm.), rat (Rn.) and zebrafish (Dr.) *CDK5R1 *3'-UTR. Stars represent fully conserved positions; the best ARE consensus is boxed in gray, the polyA sites are in bold. C) Representation of the most stable secondary structure of the human *CDK5R1 *3'-UTR as predicted by the Sfold algorithm. The magnification shows the loop (arrow) corresponding to the ARE with the best consensus (nt 2659–2671).

We previously predicted a potential GY-box and three putative AREs in *CDK5R1 *3'-UTR [19]. In order to refine the search for potential Class I/II AREs we looked for the AUUUA pentameric motif in the whole *CDK5R1 *3'-UTR sequence: we found eight pentamers, among which two are isolated and six are flanked at one or both sides by AU stretches (Fig. 1A). None of these putative AREs show AUUUA pentamers arranged in tandem, a feature of Class II AREs, which indicates that these motifs might belong to Class I. Nevertheless, only one of the eight potential AREs (nt 2659–2671) corresponds perfectly to the consensus sequence (WWWUAUUUAUUUW), according to the ARED 3.0 database definition [38], while the other possible AREs show at least two mismatches.

Furthermore, we assessed the conservation between human, mouse and rat *CDK5R1 *3'-UTRs, which have very similar lengths (2725 bp, 2779 bp and 2683 bp, respectively). The comparison, performed with the mVISTA tool (Fig. 1A), indicates that the three sequences show several regions of at least 100 bp with more than 70% identity, notably in their proximal and terminal portions, with an overall sequence identity of 76.8% and of 76.3% for the human-mouse and the human-rat pair, respectively; the most conserved portion of human *CDK5R1 *3'-UTR is the 3'-end, which has about 95% identity with both its mouse and rat counterpart. Based on this analysis, the three most terminal putative AREs (nt 2539–2551, nt 2636–2645 and nt 2659–2671) are completely conserved, while the other five show one or more mismatches. It is worth noting that, though the zebrafish *Cdk5r1 *3'-UTR, assembled by EST analysis, shows a high level of divergence with the sequences of mammals, the portion corresponding to the *CDK5R1 *3'-UTR ARE that matches the ARED consensus is nearly completely conserved, with the exception of only one nucleotide (Fig. 1B). As far as the GY-box element is concerned, there is only one mismatch between human, mouse and rat, while this element seems to be absent in zebrafish (data not shown).

As functional AREs are likely to be exposed in loops accessible to AU-rich binding proteins, we predicted the *CDK5R1 *3'-UTR secondary structure by using the Sfold RNA-folding algorithm [39]: this analysis indicated that in the most stable predicted structure only three possible AREs are accessible in a single-stranded loop, in particular the ARE that follows the ARED consensus criteria (nt 2659–2671) (Fig. 1C). In addition, this ARE is excluded from RNA duplexes even in the other predicted secondary structures, which show higher free energies (data not shown). The secondary structure analysis of the region of *CDK5R1 *3'-UTR surrounding the GY-box motif has previously been described [19].

### Human *CDK5R1 *3'-UTR decreases reporter gene activity

**Figure 2:**
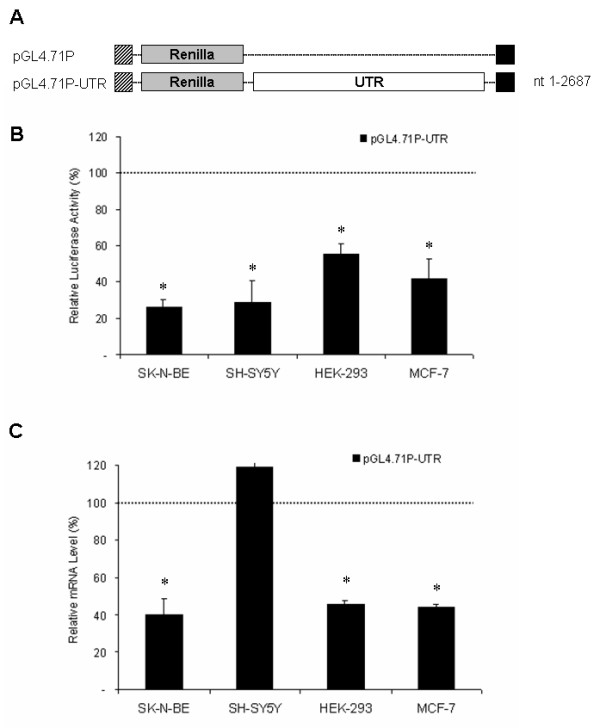
**The 3'-UTR of *CDK5R1 *causes a decrease in luciferase activity**. A) Schematic representation of luciferase construct carrying the human 3'-UTR of *CDK5R1*. The pGL4.71P control construct does not contain *CDK5R1 *UTR sequences. The Renilla luciferase gene is indicated by the grey bars, and the region of the 3'-UTR is represented by the white bars. The numbers on the right indicate the 3'-UTR nucleotides included in the chimeric construct. Numbering starts from the first nucleotide beyond the stop codon for *CDK5R1*. Transcription was under the control of the SV40 promoter (hatched bars) and the polyA site (black bars). B) Luciferase activity of pGL4.71P-UTR construct in SK-N-BE, SH-SY5Y, HEK-293 and MCF-7 cell lines assessed using the Dual-Glo Luciferase assay system. Cells were transiently co-transfected with the pGL4.71P-UTR construct (Renilla luciferase) and the pGL3 (Firefly luciferase) vector and were harvested 24 hours post-transfection (see Methods). Luciferase activity of the chimeric reporter gene, normalized for transfection efficiency against the Firefly luciferase activity, is represented as a percentage of the activity observed in cells transfected with pGL4.71P (defined as 100%). Means ± s.d. luciferase values were obtained from at least four independent experiments (* p < 0.01 compared with the corresponding pGL4.71P value). C) mRNA levels of the chimeric transcript. Total RNA was extracted from the cells harvested 24 hours post-transfection, and the reporter gene mRNA levels were analyzed by RealTime PCR as described. mRNA levels of the chimeric reporter gene, normalized against the housekeeping gene *GAPDH *and for transfection efficiency against the Firefly luciferase mRNA levels, are represented as a percentage of the mRNA levels observed in cells transfected with pGL4.71P (defined as 100%). Means ± s.d. luciferase mRNA values were obtained from at least three independent experiments (* p < 0.01 compared with the corresponding pGL4.71P value).

The effect of the 3'-UTR on gene expression was studied with the luciferase reporter assay. A construct was generated carrying the human *CDK5R1 *3'-UTR downstream of the reporter gene (Fig. 2A).

The construct pGL4.71P-UTR was transiently transfected in neuroblastoma cell lines SK-N-BE and SH-SY5Y, in human embryonic kidney HEK-293 cells and breast tumor MCF-7 cell lines. Luciferase activity was measured 24 hours after transfection.

The ARE region of *Bcl2 *gene from nt 942 to 1020 (GenBank M14745) has been used as a positive control (construct pGL4.71P-b-ARE) as it was shown to have a degradation activity on the mRNA [40]. pGL4.71P-b-ARE leads to a decrease in luciferase activity in all four cell lines (data not shown).

The luciferase activity of construct pGL4.71P-UTR was significantly lower in the four transfected cell lines compared to the insertion-less pGL4.71P vector (p < 0.01) (Fig. 2B). The amount of reporter RNAs in the cell lines transfected with the above luciferase constructs was quantified by RealTime RT-PCR in order to identify whether the decrease in luciferase activity was due to lower mRNA levels. Data were normalized to Firefly to control for transfection efficiency and for the housekeeping gene *GAPDH*. The levels of mRNA for each sample correlated with the luciferase activity, with the exception of SH-SY5Y cells where the transcript level is similar to the insertion-less pGL4.71P vector (Fig. 2C).

### *CDK5R1 *3'-UTR fragments differently affect reporter gene expression

**Figure 3:**
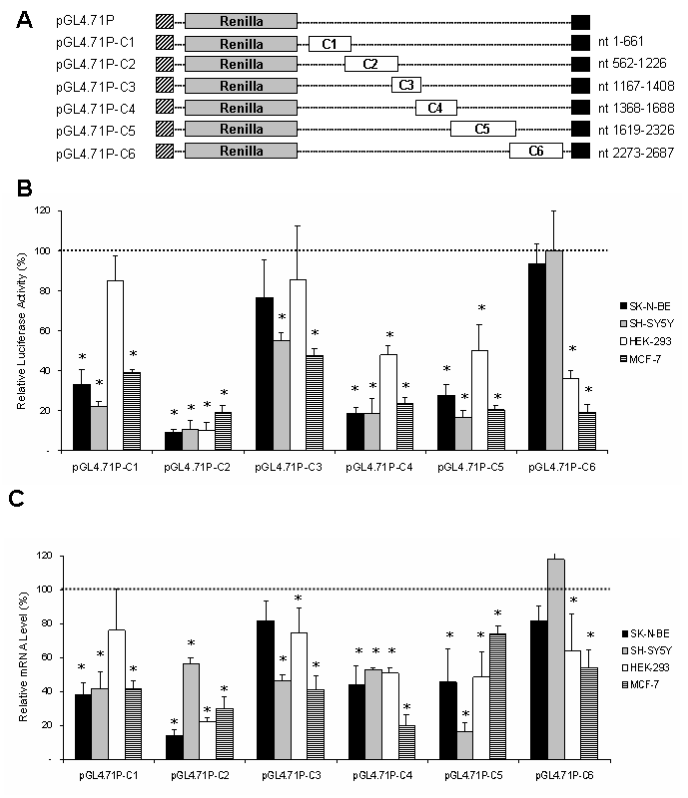
***CDK5R1 *3'-UTR fragments differently affect reporter gene expression**. A) Schematic representation of luciferase constructs carrying fragments of the 3'-UTR of *CDK5R1*. The pGL4.71P control construct contained no *CDK5R1 *UTR sequences. The chimeric constructs were created by cloning C1, C2, C3, C4, C5 and C6 3'-UTR fragments downstream of the Renilla luciferase gene. B) Luciferase activity of the six pGL4.71P- constructs in SK-N-BE, SH-SY5Y, HEK-293 and MCF-7 cell lines. Cells, transiently co-transfected with the pGL4.71P- constructs (Renilla luciferase) and the pGL3 (Firefly luciferase) vector were harvested 24 hours post-transfection. Luciferase activity of the chimeric constructs normalized as described, is represented as a percentage of the activity observed in cells transfected with pGL4.71P (defined as 100%). Means ± s.d. luciferase values were obtained from at least four independent experiments (* p < 0.01 compared with the corresponding pGL4.71P value). C) mRNA levels of the chimeric transcripts. Total RNA was extracted from the cells harvested 24 hours post-transfection, and the reporter gene mRNA levels were analyzed by RealTime PCR. mRNA levels of the chimeric reporter gene normalized as described, are represented as a percentage of the mRNA levels observed in cells transfected with pGL4.71P (defined as 100%). Means ± s.d. luciferase mRNA values were obtained from at least three independent experiments (* p < 0.01 compared with the corresponding pGL4.71P value).

In order to study whether the destabilizing effect on luciferase activity can be assigned to specific regions, the 3'-UTR was divided into six smaller fragments (C1-C6) each containing at least one putative regulatory element according to the *in silico *prediction described above, and six luciferase reporter constructs were generated (Fig. 3A).

For each construct we performed a dual luciferase assay as described above. Fragmentation of the 3'-UTR shows that pGL4.71P-C2, pGL4.71P-C4 and pGL4.71P-C5 lead to a considerable decrease in luciferase activity in all cell lines (p < 0.01) indicating the presence of destabilizing regulatory elements (Fig. 3B). In particular, pGL4.71P-C2 has the strongest decrease of luciferase activity observed, by more than 80% compared to the control construct. The other fragments decrease luciferase activity with significant difference among cell lines, showing destabilization in all lines with exception of HEK-293 for pGL4.71P-C1 and SK-N-BE and HEK-293 for pGL4.71P-C3. pGL4.71P-C6 decreases luciferase activity only in HEK-293 and MCF-7 cells (p < 0.01) and was not effective in neuroblastoma derived lines.

The quantitation of the above luciferase constructs mRNAs in SK-N-BE, SH-SY5Y, HEK-293 and MCF-7 cell lines by RealTime RT-PCR correlated in most cases with the luciferase activity. Exceptions were the constructs pGL4.71P-C2 in SH-SY5Y, pGL4.71P-C4 in SK-N-BE and SH-SY5Y and pGL4.71P-C5 in MCF-7 in which the reduction of mRNA levels was not so marked as the correspondent luciferase activity (Fig. 3C). These data were normalized as previously described.

### C2 fragment increases the rate of degradation of the reporter mRNA in an ARE independent way

As the pGL4.71P-C2 construct led to the strongest reduction of luciferase activity and mRNA level (Fig. 3) we performed a degradation assay in order to determine whether accelerated mRNA decay can contribute to the observed reduction in luciferase activity and mRNA in SK-N-BE cells.

**Figure 4:**
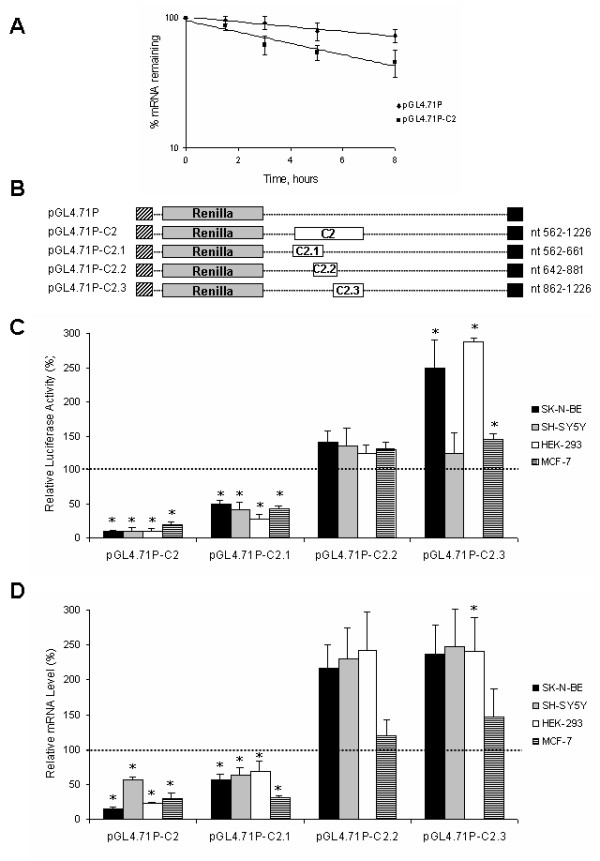
**Decrease in steady state mRNA levels is due to altered rates of messenger degradation which is not ARE dependent**. A) Decay of pGL4.71P-C2 mRNA (black squares) compared with that of pGL4.71P control (black diamonds). SK-N-BE cells were transiently transfected with reporter gene constructs and the pGL3 (firefly luciferase) vector. 24 hours after transfection (time = 0 h), cells were treated with DRB. Total RNA was extracted after various time-points. RNA was reverse-transcribed and RealTime RT-PCR was performed. Renilla luciferase transcript levels were normalized to GAPDH and firefly luciferase mRNAs. Data are expressed as percentage of RNA remaining after DRB addition and are representative of three independent experiments. B) Schematic representation of luciferase constructs carrying C2 fragment and sub-fragments. C) Luciferase activity of the chimeric constructs in SK-N-BE, SH-SY5Y, HEK-293 and MCF-7 cell lines. Cells, transiently co-transfected with the pGL4.71P- constructs (Renilla luciferase) and the pGL3 (Firefly luciferase) vector were harvested 24 hours post-transfection. Luciferase activity of the chimeric constructs normalized as described, is represented as a percentage of the activity observed in cells transfected with pGL4.71P (defined as 100%). Means ± s.d. luciferase values were obtained from at least four independent experiments (* p < 0.01 compared with the corresponding pGL4.71P value). D) mRNA levels of the chimeric transcripts. Total RNA was extracted from the cells harvested 24 hours post-transfection, and the reporter gene mRNA levels were analyzed by RealTime PCR. mRNA levels of the chimeric reporter gene normalized as described, are represented as a percentage of the mRNA levels observed in cells transfected with pGL4.71P (defined as 100%). Means ± s.d. luciferase mRNA values were obtained from at least three independent experiments (* p < 0.01 compared with the corresponding pGL4.71P value).

We performed a real-time quantitative RT-PCR to compare mRNA levels between the pGL4.71P-C2 and pGL4.71P in SK-N-BE cells at various times after the addition of the transcriptional inhibitor DRB, which was initially added 24 hours following transfection. These data were normalized on transfection efficiency by using Firefly mRNA levels and GAPDH expression as housekeeping gene. With respect to mRNA decay, the calculated half-life of the pGL4.71P-C2 construct was 5,73 h, significantly lower compared to the insertion-less construct pGL4.71P- being > 8 h (Fig. 4A). These findings suggest that an accelerated degradation of mRNA has contributed to the observed reduction in reporter gene expression and mRNA associated with the C2 fragment.

In order to restrict the region causing the accelerate degradation of C2 fragment and to determine the role of the potential nt 992–1001 ARE motif, we divided the C2 fragment, according to the presence of conserved stretches, into 3 sub-fragments named C2.1 (138 bp), C2.2 (240 bp) and C2.3 (365 bp) (Fig. 4B) the latter containing the nt 992–1001 ARE. This fragments were cloned in the above described reporter vector.

For each construct we performed a dual luciferase assay and the quantization of the luciferase constructs mRNAs as described above. Fragmentation of C2 shows that only pGL4.71P-C2.1 leads to a considerable decrease in luciferase activity and mRNA level in all cell lines (p < 0.01) indicating the presence of unknown regulatory element/s with a destabilizing effect (Fig. 4C and 4D). It is worth to be noted that the C2.3 construct, containing the putative ARE, does not reduce both luciferase and mRNA levels.

### Class I ARE (nt 2659–2671) has a stabilizing effect in neuroblastoma cells while GY-box element does not affect reporter gene expression

We focused on the only canonical ARE (nt 2659–2671), localized in the C6 fragment, which has the consensus sequence for a class I ARE, and on the GY-box element, localized in the C3 fragment.

**Figure 5:**
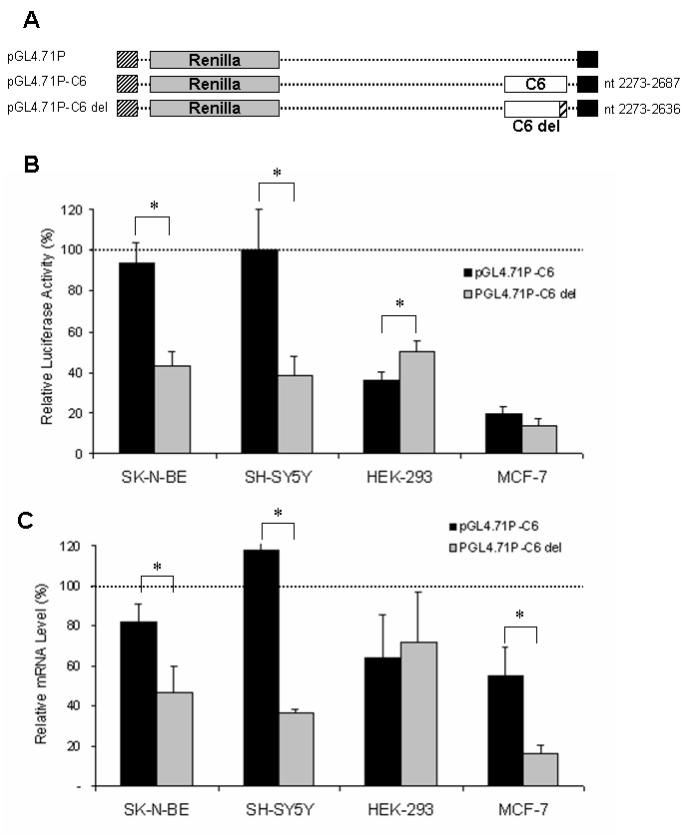
**Effect of deletion of the canonical Class I ARE**. A) Schematic representation of control and deleted constructs. The region containing Class I ARE element (nt 2637–2687) was deleted (hatched bar) from the pGL4.71P-C6 in construct pGL4.71P-C6del by PCR. B) Luciferase activity of the two chimeric constructs in SK-N-BE, SH-SY5Y, HEK-293 and MCF-7 cell lines. Cells, transiently co-transfected with the pGL4.71P- constructs (Renilla luciferase) and the pGL3 (Firefly luciferase) vector were harvested 24 hours post-transfection. Luciferase activity of the chimeric constructs normalized as described, is represented as a percentage of the activity observed in cells transfected with pGL4.71P (defined as 100%). Means ± s.d. luciferase values were obtained from at least four independent experiments (* p < 0.01 compared with the corresponding pGL4.71P value). C) mRNA levels of the chimeric transcripts. Total RNA was extracted from the cells harvested 24 hours post-transfection, and the reporter gene mRNA levels were analyzed by RealTime PCR. mRNA levels of the chimeric reporter gene normalized as described, are represented as a percentage of the mRNA levels observed in cells transfected with pGL4.71P (defined as 100%). Means ± s.d. luciferase mRNA values were obtained from at least three independent experiments (* p < 0.01 compared with the corresponding pGL4.71P value).

To verify the actual function of the putative class I ARE, the region from nt 2637 to 2687, encompassing the ARE element, was deleted from C6 fragment (Fig. 5A). As shown in Fig. 5B, pGL4.71P-C6del luciferase activity is lower in comparison with that of the insertion-less plasmid (p < 0.01) in all four cell lines. It is worth noting that in SK-N-BE and SH-SY5Y lines, the mRNA level of the pGL4.71P-C6del construct decreases in sharp contrast with the non-deleted construct (p < 0.01) indicating that the class I ARE might act as a stabilizing element in these cell lines (Fig. 5C).

Regarding the GY-box, the sequence GTCTTCC (nt 1341–1347) was deleted from the C3 fragment. Normalized luciferase activity was not affected by the constructs with deleted GY-box in comparison with the corresponding non deleted construct in all the tested cell lines (data not shown), indicating the inactivity of this element in the cell lines used.

### *CDK5R1 *mRNA is a target of nELAV RNA-binding proteins

**Figure 6:**
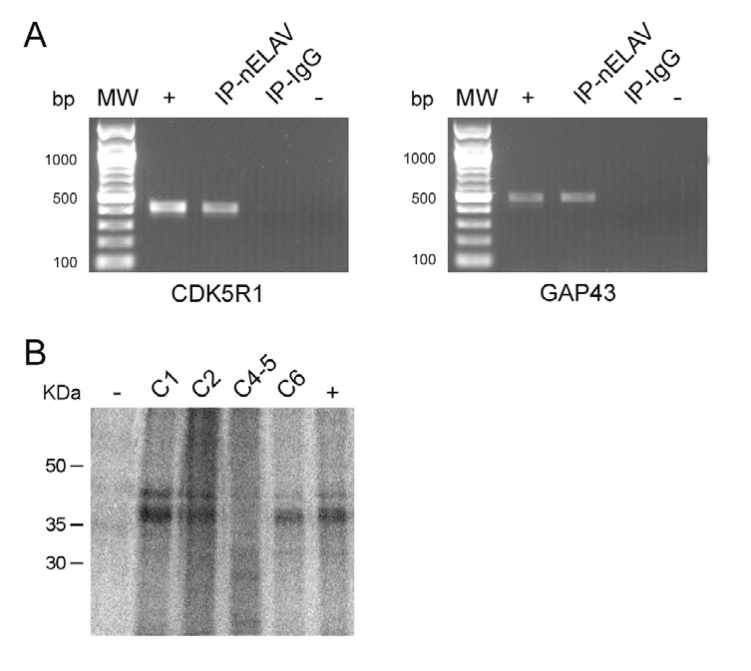
***CDK5R1 *mRNA is bound by nELAV proteins in SH-SY5Y cells**. A) Endogenous mRNP complexes were selectively immunoprecipitated from SH-SY5Y cells using the pan neuronal ELAV and the irrelevant IgG antibodies. The bound mRNAs were analyzed by RT-PCR with primer pairs specific for *CDK5R1 *(left panel) and GAP-43 (right panel). MW, Molecular weight marker; +, positive control (total SH-SY5Y cDNA); -, negative control (no sample). B) In vitro RNA-protein binding experiments were performed by UV cross-linking of different P^32^-radiolabelled regions from *CDK5R1 *3'UTR and brain protein lysate. A selective immunoprecipitation of the formed mRNP complex was performed using the nELAV antibody (lanes 2–5). As a negative control a mix of all the different riboprobes was used and immunoprecipitated by the IgG antibody (-, lane 1), while GAP-43 3'UTR riboprobe was used as a positive control (+, lane 5).

Since *CDK5R1 *3'UTR contains several putative ARE sequences and the ARE-containing C6 fragment seems to confer mRNA stability specifically in the neuronal SK-N-BE and SH-SY5Y cell lines, we investigated whether neuronal RNA-binding proteins (RBPs) are involved in the post-transcriptional control of *CDK5R1 *mRNA. Among the ARE-binding proteins the ELAV family is of interest since three members (HuB, HuC, HuD) out of four are selectively expressed in neurons and display a stabilizing effect on target mRNAs. We tested the binding of the nELAV RBPs to the *CDK5R1 *transcript by immunoprecipitating endogenous mRNA-protein (mRNP) complexes from SH-SY5Y cells with the pan-neuronal ELAV antibody. The presence of *CDK5R1 *transcript together with *GAP43*, which is a well-known target of nELAV proteins, was detected by RT-PCR amplification of co-precipitated mRNAs (Fig. 6A). We also used the irrelevant IgG antibody as a negative control in the assay to confirm that the *CDK5R1 *mRNA was selectively isolated only from nELAV-immunoprecipitated mRNP complexes.

In order to map the binding of nELAV proteins along the *CDK5R1 *transcript, we dissected the 3'UTR into four sub-regions corresponding to the ARE containing fragments C1, C2, C4-5 and C6, and used them in *in vitro *UV cross-linking experiments. The radio-labelled C1, C2, C4-5 and C6 riboprobes were incubated in the presence of brain protein lysates and, after UV irradiation, the formed mRNP complexes were immunoprecipitated by the nELAV antibody and resolved on SDS-PAGE. We observed that nELAV RBPs recognized and bound C1, C2 and C6 sub-regions, while the C4-5 fragment did not form mRNP complexes with nELAV proteins (Fig. 6B).

## Discussion

*CDK5R1 *is known to be a key regulator of central nervous system development and functioning [1] and it has recently been shown to have several extra-neuronal roles [20]. *CDK5R1 *3'-UTR is longer than the eukaryotic 3'-UTR average [24], belonging to the 5% longest annotated 3'UTRs [21] and was recently reported to contain potential post-transcriptional regulatory elements [19], suggesting that it may be important for the control of *CDK5R1 *gene expression. The particular features of *CDK5R1 *3'-UTR and the lack of functional data prompted us to study its role in the control of gene expression by means of bioinformatic analysis and dual luciferase and RealTime PCR assays of chimeric constructs.

We have shown that the *CDK5R1 *3'-UTR can down-regulate luciferase expression and we assessed the presence of different sub-regions that can independently affect transcript stability and, in a few cases, translational efficiency in different cell lines.

The high degree of conservation showed by *CDK5R1 *3'-UTR is strongly indicative of a functional role of this region and suggests the presence of different post-transcriptional regulatory elements [41]. In particular several AREs were predicted, a feature typical of mRNAs with high turnover rate [42], among which the nt 2659–2671 ARE shows complete identity to the ARE consensus sequence, according to the ARED 3.0 definition. Together with the high phylogenetic conservation in mammals and zebrafish and with the predicted accessibility to the binding of trans-acting factors, the functionality of this element can be hypothesized. It is worth noting that functional AREs are present in the 3'-UTR of many cyclin mRNAs [43], raising the hypothesis that ARE-mediated post-transcriptional regulation may be a common mechanism for the control of expression in this class of genes, to which *CDK5R1 *belongs.

The pGL4.71P-UTR construct shows a decreased luciferase activity in all the cell lines according to mRNA level with the exception of SH-SY5Y cells, showing an mRNA level comparable to that of the control, which could be explained by the action of mechanisms controlling the translation. The overall effect of the large *CDK5R1 *3'-UTR may be the result of a complex regulation mechanism mediated by multiple 3'-UTR domains that act independently of each other, as has been already highlighted in *BCL2*, *Cox-2*, *VEGF *and *c-Fos *mRNAs [40,44-48]. The dissection of 3'-UTR into 6 fragments, each containing at least one predicted regulatory element, allowed us to investigate the potential role of each region. All the chimeric constructs showed, in most of the studied cell lines, a general decrease of luciferase activity. In most cases these effects are likely to involve transcript stability rather than translational repression mechanisms, since reduced reporter activity levels corresponded, as determined by RealTime RT-PCR experiments, to diminished mRNA levels. In some instances the reduction of luciferase activity may be the result of mechanisms affecting both mRNA stability and translational efficiency. This effect is particularly evident for C2 and 3'-UTR constructs in SH-SY5Y cell line, suggesting that some elements influencing the behavior of the entire *CDK5R1 *3'-UTR may be found in the C2 region (Fig. 2 and 3).

The strong reduction of luciferase activity and RNA induced by C2 fragment in all the transfected cell lines suggests the presence of one or more regulatory elements with a potent destabilizing function supported by the degradation assay results. We found that the C2.3 fragment, containing the only known putative element (nt 992–1001 ARE), is not responsible for the low expression of the C2 fragment and thus this ARE, which is not conserved in rodents, is unlikely to be a destabilizing element. On the other hand, the decreased expression of pGL4.71P-C2.1 construct, in which none putative regulatory elements are predicted, prompts us to hypothesize the presence of novel regulatory elements in the C2.1 fragment. Further studies will be necessary to identify the post-transcriptional mechanisms leading to mRNA degradation of the above construct.

The non-destabilizing effect detected in HEK-293 cells for the C1 fragment and in SK-N-BE and SH-SY5Y for C6 allows us to hypothesize that these regions are bound by cell-line specific stabilizing factors. The deletion in the C6 fragment of the canonical nt 2659–2671 ARE reduced mRNA levels in all the analyzed cell lines, including SK-N-BE and SH-SY5Y. This finding strongly suggests a stabilizing role of the canonical element in neuroblastoma-derived cells through the binding of neuronal-specific stabilizing factors expressed in SK-N-BE and SH-SY5Y cells (Fig. 4). The prediction that this ARE is within a single-stranded loop further supports this hypothesis. Several ARE binding proteins are known, some of which promote the stabilization of their target mRNAs, including cyclin mRNAs [24,43]. Among these proteins, the neuronal specific nELAV RBPs, HuB, HuC and HuD, play a key role in the induction of neuronal differentiation [49-51] and have a stabilizing effect on several transcripts containing AREs in their 3'-UTR [52,53]. The expression of HuB and HuD has been demonstrated in several neuroblastoma cell lines [54].

Our immunoprecipitation assays show that the neuronal-specific nELAV RBPs bind to *CDK5R1 *transcript in SH-SY5Y cells. The UV cross-linking experiments allowed us to observe that C1, C2 and C6 sub-regions show affinity for nELAV proteins *in vitro *while, although C4-5 contains putative AREs, it does not bind to nELAV RBPs. The luciferase activity together with the RealTime PCR results in comparison to UV cross-linking data, suggest that the binding shown by C1 and C2 regions for nELAV may be not so effective *in vivo *as in the case of C6 region. This finding might be explained by possible interactions of these regions with further regulatory destabilizing factors or to possible formation of unfavourable secondary mRNA structures. According to the RNA-operon model, trans-acting factors regulate mRNAs within a coordinated pathway of RNA processing, providing cells of a fast and dynamic tool to respond to environmental cues [55].

Our results on interaction of the nELAV factors with *CDK5R1 *messenger in a neuronal cell line support the hypothesis of a neuron-specific post-transcriptional regulation of *CDK5R1 *expression. This mechanism may be activated during central nervous system development to rapidly increase dosage of a protein such as p35, characterized by a rapid turnover through the ubiquitin-proteasome pathway [56].

The generation of a construct with the canonical GY-box motif deleted revealed the inactivity of this element in all the cell lines used for the transfection experiments. These data indicate that this motif does not seem to be involved in the regulation of *CDK5R1 *expression.

Our findings have shown the presence of several regulatory elements in *CDK5R1 *3'-UTR, and for a few of them we assessed a destabilizing or stabilizing function. The 3'-UTR seems to contain some regulatory elements implicated in rapid mRNA turnover which, as a consequence, maintain the steady-state transcript at low levels, and others which have a cell-specific stabilizing effect on the transcript that may contribute to rapidly increase the expression of *CDK5R1 *during specific biological processes. Thus the 3'-UTR may exert a key role in fine tuning of *CDK5R*1 expression that in turn might be crucial during the complex processes underlying correct central nervous system development and neuronal survival in which *CDK5R1 *is involved [12,57]. The active CDK5-p35 complex is strongly involved in neurofilament phosphorylation and its aberrant hyperactivity has been shown to be implicated in neurodegenerative diseases [58]. It has recently been demonstrated in mouse that p35 protein level is a rate-limiting factor for the up-regulation of Cdk5 activity [59]. Thus an up-regulation of *CDK5R1 *expression in neurons may have pathological implications causing neurodegenerative diseases. On the other hand, the absence of p35 leads to abnormalities in the laminar structure of cerebral cortex, affecting actin-cyotskeletal dynamics and microtubule regulation [60], and severe mental retardation was shown by patients with NF1 microdeletion syndrome with the lack of one copy of *CDK5R1 *[18]. According to our results we hypothize that an up- or down-regulation of *CDK5R1 *expression, caused by defective post-transcriptional mechanisms, may have pathogenetic implications in neurodegenerative disorders or cognitive impairment-based diseases, respectively.

Despite the fact that p35 activity has primarily been associated with central nervous system, several extra-neuronal roles of p35 have recently been shown, causing direct or indirect effects on the organization of cytoskeletal structures [20]. The *CDK5R1 *expression is likely to be finely controlled also in non-neuronal tissues. Our findings on non-neuronal cell lines suggest that the 3'-UTR might be involved in the post-transcriptional control of *CDK5R1 *expression in different tissues and that an alteration of this mechanism may cause cellular dysfunction and disease. The large 3'-UTR of *CDK5R1 *is expected to contain further regulatory elements, creating the possibility of complex gene expression modulation [61].

## Conclusion

Our study evidences for the first time the involvement of 3'-UTR in the modulation of *CDK5R1 *expression by the presence of both destabilizing and stabilizing cis-regulatory elements in 3'-UTR, also supporting the hypothesis that *CDK5R1 *gene expression is controlled in neurons by nELAV-mediated mechanisms, with potential implications in neurodegenerative and cognitive disorders.

Additional studies are necessary to validate the biological function of the predicted regulatory elements and to identify novel cis-acting regulatory motifs such as microRNA target sites, in order to evince the role of 3'-UTR in the regulation of *CDK5R1 *expression. This search may eventually clarify the molecular basis of some neurological diseases and in perspective the validated regulatory elements may represent new pharmacological targets for treatment of neurodegenerative diseases.

## Methods

### Bioinformatic analysis

The human and mouse *CDK5R1 *3'-UTR sequences (3HSA086450 and 3MMU05479) were obtained from the UTRdb database [62] and correspond to the 3'-UTR of the NCBI Reference Sequence cDNAs NM_003885 and NM_009971, respectively [63]. The rat and zebrafish *Cdk5r1 *3'-UTR sequences were assembled starting from the ESTs annotated in the UCSC Genome Browser (rat assembly version 3.4 (rn4) and zebrafish assembly version 6 (Zv6)) using the CAP3 sequence assembly program [64] with default parameter values. The AREs were searched using the PatSearch program [65], looking for the AUUUA pentamer; the results were compared with the annotation proposed by the ARED 3.0 database [38]. Comparative analysis was carried out using the LAGAN alignment program of mVISTA [66]. and the ClustalX program [67]. The Sfold software was used for the prediction of *CDK5R1 *3'-UTR secondary structure [39].

### 3'-UTR constructs

Segments of the human *CDK5R1 *3'-UTR were PCR-amplified using primers containing flanking *Xba*I recognition sequence (Table 1). The segment in the 3'-UTR from nucleotide 1167 to 1408 carrying the deletion of the GY-box element, named C3del, was obtained by nested deletion of the GY-box element (GCTCCTT) from nucleotide 1341 to 1347. Two partially overlapping PCR products were synthesized for this purpose, as described by [68]. Briefly, the first PCR product was amplified with C3 FW (see Table 1) and 5'-*GGGCCT*AGAATCCTCTGTAGTGTCTTCA-3' GYdel reverse primers. The second PCR product was amplified with 5'-*AGGCCC*AGTCCACTGGGGA-3' (GYdel FW) and C3 REV primers. The 6 overlapping nucleotides are italicized in GYdel REV and GYdel FW primers. The PCR products were ligated in the *Xba*I restriction site downstream of the Renilla luciferase coding region of the pGL4.71 vector (Promega, Madison, WI) in which the SV40 promoter region from the pGL3-Promoter vector (Promega) was previously cloned to obtain the pGL4.71P plasmid. Correct orientation of the insert was verified by sequencing.

**Table 1:** Oligonucleotides used to generate constructs of the 3'-UTR of the human CDK5R1 gene.

**Fragment**	**nt (bp)**	**Sequence**	**PCR annealing (°C)**
UTR	1–2687	Fw: 5' GC TCTAGAATCGGTGAGCACTGTGCCTG 3' Rev: 5' GCTCTAGAGAATCAGCACAGTACAAAAATAAAT 3'	62
C1	1–661	Fw: 5' GCTCTAGAATCGGTGAGCACTGTGCCTG 3' Rev: 5' GCTCTAGAAGCAGCAGACAAGGGGGTAG 3'	62
C2	562–1226	Fw: 5' GCTCTAGATGAGCGGGTCTAGTGGAAAG 3' Rev: 5' GCTCTAGAGAAAGAAAATCAATAAAGTACAC 3'	58
C2.1	562–661	Fw: 5' GCTCTAGATGAGCGGGTCTAGTGGAAAG 3' Rev: 5' GCTCTAGAAGCAGCAGACAAGGGGGTAG 3'	58
C2.2	642–881	Fw: 5' GCTCTAGACTACCCCCTTGTCTGCTGCT 3' Rev: 5' GCTCTAGAGTATGGCATCCCTCACCTTG 3'	58
C2.3	862–1226	Fw: 5' GCTCTAGACAAGGTGAGGGATGCCATAC 3' Rev: 5' GCTCTAGAGAAAGAAAATCAATAAAGTACAC 3'	56
C3	1167–1408	Fw: 5' GCTCTAGAGTTTCCACCTTACCCTACTG 3' Rev: 5' GCTCTAGAAGTGACCCTCTGCCTCCCC 3'	58
C4	1368–1688	Fw: 5' GCTCTAGATGCTGGAATAGGGACCTGG 3' Rev: 5' GCTCTAGAGTGCTGTGTGAAGTCTGTG 3'	56
C5	1619–2326	Fw: 5' GCTCTAGAGGGACTGTCAGATAATCGGTG 3' Rev: 5' GCTCTAGATCCAGGTTTACAAGAAAAAGAGAA 3'	62
C6	2273–2687	Fw: 5' GCTCTAGACCACAGGAATAATAGTTCAGG 3' Rev: 5' GCTCTAGAGAATCAGCACAGTACAAAAATAAAT 3'	56
C6del	2273–2636	Fw: 5' GCTCTAGACCACAGGAATAATAGTTCAGG 3' Rev: 5' GCTCTAGACAGCCAAACTGAGCTTCATG 3'	58

XbaI recognition site sequence is underlined

### Cell Cultures

Human neuroblastoma SK-N-BE cells were cultured in RPM-I medium with 10% fetal calf serum (FCS), 100 U/ml penicillin-streptomycin, 0.01 mM L-glutamine, sodium pyruvate 11 g/l and glucose 4.5 g/l. Human neuroblastoma SH-SY5Y cells and human embryonic kidney HEK-293 cells were maintained in DMEM medium with 10% FCS, 100 U/ml penicillin-streptomycin and 0.01 mM L-glutamine. Human breast tumor MCF-7 cells were cultured in RPM-I medium with 10% FCS, 100 U/ml penicillin-streptomycin and 0.01 mM L-glutamine (all media ingredients were obtained from Sigma-Aldrich, AS, Oslo, Norway). Cultures were maintained at 37°C in a 5% CO_2 _incubator.

### Cell transfection

For luciferase activity assays, 2.5–4.5 × 10^6 ^cells were plated in 6-well dishes in 2 ml of medium 24 hours before transfection. Cells were transfected at 60–70% confluence with 300 ng of the pGL4.71P constructs containing different *CDK5R1 *3'-UTR fragments. To normalize the value of Renilla luciferase activity for transfection efficiency and cell viability after transfection, the pGL3-Promoter Firefly luciferase reporter gene was co-transfected (300 ng). For transfection 2 μl of LIPOFECTAMINE 2000 (Invitrogen, Burlington, ON) were used according to the manufacturer's instructions.

### Measurement of luciferase activity

Luciferase reporter assays were performed using the Dual-Glo Luciferase Reporter Assay System (Promega). 24 hours after transfection, the culture medium was removed, and cells were washed with phosphate-buffered saline. Passive Lysis Buffer (350 μl of 1× buffer) was added to each well, and plates were placed in a shaking incubator for 30 minutes at room temperature. For additional lysis, two freeze-thaw cycles were performed in which the cells were frozen to -80°C. 50 μl of Dual-Glo Luciferase Reagent were added to 50–100 μl of lysate and incubated for 30 minutes. Firefly luciferase luminescence (pGL3P vector) was measured. Before measurement of Renilla luciferase (pGL4.71P vector), 50 μl of the Stop & Glo reagent were added to each well to quench the firefly luciferase reaction. Renilla luciferase luminescence was measured after an incubation of 30 minutes. Relative Renilla luciferase light output was normalized to Firefly luciferase output. Firefly-normalized luciferase activity for the construct was compared with the insertion-less pGL4.71P vector. The data were expressed as means ± s.d. Statistical significance was calculated by using a Student's t test. A difference between two means was considered statistically significant when p < 0.01.

### Total RNA extraction, cDNA synthesis, and quantitative PCR

For total mRNA assays, 0.85–1.5 × 10^6 ^cells were plated in a 25 cm^2 ^flask in 4 ml of medium 24 hours before transfection. Cells were co-transfected at 60–70% confluence with 500 ng pGL3P and 500 ng of pGL4.71P construct containing different *CDK5R1 *3'-UTR fragments, using 4 μl of Lipofectamine 2000 (Invitrogen) according to the manufacturer's instructions. Total RNA was extracted using NucleoSpin RNA II Columns (Macherey-Nagel, Düren, Germany) according to manufacturer's instructions. To eliminate amplification of reporter plasmid DNA and genomic DNA, total RNA was treated with Turbo DNA-free (Ambion Inc., Austin, TX) at 37°C for 30 minutes. RNA (4 μg) was reverse transcribed with High Capacity cDNA Archive Kit (Applied Biosystems Inc., Foster City, CA). The level of luciferase transcripts was determined using RealTime quantitative reverse transcriptase PCR using the TaqMan system (Applied Biosystems Inc.) using specific primers and probes for Renilla luciferase, Firefly luciferase and Glyseraldehyde-3-phosphate dehydrogenase (*GAPDH*). Renilla and Firefly luciferase quantification data were normalized on expression of the housekeeping gene *GAPDH*, and then the Renilla data were normalized on transfection efficiency by using Firelfy mRNA levels.

In mRNA decay assays SK-N-BE cells were treated with 5,6-dichloro-1-beta-D-ribobenzimidazole (DRB) (1 μg/ml) 24 hours post-transfection to suppress the transcription of the luciferase reporter gene. Cells were harvested at 0, 1.5, 3, 5 and 8 hours after treatment with DRB and RNA isolated for real time RT-PCR analysis.

### mRNP isolation and immunoprecipitation

Isolation of endogenous mRNPs was conducted with minor modifications to [69] and as already described [53]. Briefly, 2 × 10^6 ^SH-SY5Y cells were harvested for each experiment and resuspended in RNP buffer (100 mM KCl, 5 mM MgCl_2_, 10 Mm HEPES pH 7.4, 0.5% NP-40). Lysates were incubated in 1 ml NT2 buffer (50 mM Tris-HCl pH 7.4, 150 mM NaCl, 1 mM MgCl_2_, 1 mM DTT, 20 mM EDTA, 0.05% NP-40) together with protein G Sepharose-beads (GE Healthcare, Fairfield, CT) pre-coated with 4 μg of pan neuronal ELAV (16A11, Invitrogen) or Ig-G (Santa Cruz Biotechnology, Santa Cruz, CA) antibodies. RNA was phenol-cloroform extracted from immunoprecipitated mRNPs after treatment with proteinase K for 30 minutes. After DNaseI digestion, the isolated mRNAs were retro-transcribed using SuperScriptII RT (Invitrogen) and oligodT primers. RT-PCR was performed in two-rounds amplifications (20 cycles for the first round and 20–25 cycles for the second one) with primers pairs for *CDK5R1 *(For CCACAGGAATAATAGTTCAGG; Rev GAATCAGCACAGTACAAAAATA) and GAP43 (For TGATGCTGCCACAGAGCAGG; Rev TGGGAAAGGACAGACTCACAGACGTG). Amplifications products were run on 1% agarose gel in TAE buffer.

### In vitro transcription and UV cross-linking

Radio-labelled riboprobes were obtained by transcribing 0.5 μg restriction enzyme-linearized construct DNA with 20 U T7 RNA polymerase (Roche, Basel, Switzerland), 20 μCi α-[^32^P]UTP, 0.5 mM NTPs, and 20 U RNase inhibitor (Promega). After template DNA removal by DNaseI digestion, the resulting ^32^P-labelled riboprobes were purified on ProbeQuant G-50 microcolumns (GE Healthcare). UV cross-linking and immunoprecipitation experiments were performed as previously described [53] using the pan neuronal ELAV antibody (16A11, Invitrogen), which recognizes the three neuronal ELAV proteins but not HuR. 300,000 cpm of riboprobes were incubated with 40 μg of brain protein extract in 15 μl ligation buffer (1.3 mM MgCl_2_, 19 mM HEPES-KOH pH 7.4, 1.5 mM ATP, 19 mM Creatine phosphate) for 10 minutes at 30°C. After addition of 5 μg tRNA, samples were irradiated with UV light (Stratalinker^®^, Stratagene) for 5 minutes and RNaseA-treated (25 U) for 30 minutes. Immunoprecipitation was conducted on UV cross-linked samples by the addition of 4 μg of the selected antibody pre-coated to protein G Sepharose-beads. Immunocomplexes were then collected by centrifugation at 14,000 × g for 30 seconds, washed several times in NT2 buffer, run on a 10% SDS-PAGE and analyzed by autoradiography.

## List of abbreviations

The abbreviations used are: *CDK5R1*, *cyclin-dependent kinase 5 regulatory subunit 1*; 3'-UTR 3'untraslated region; ARE, AU-rich element; *CDK5, cyclin-dependent kinase 5*; FCS, fetal calf serum; HEK-293, Human embryonic kidney 293; DMEM, Dulbecco's modified Eagle's Medium; RT-PCR, reverse transcriptase-polymerase chain reaction; *BCL-2, B-cell lymphoma/leukemia-2*;*GAPDH, Glyseraldehyde-3-phosphate dehydrogenase*; DRB, 5,6-dichloro-1-beta-D-ribobenzimidazole; *Cox-2, cytochrome c oxidase II*; *VEGF, vascular endothelial growth factor*; *c-Fos, cellular FBJ murine osteosarcoma viral oncogene homolog*; nELAV, neuronal-specific embryonic lethal, abnormal vision; RBP, RNA-binding protein.

## Authors' contributions

SM generated and sequenced chimeric constructs, carried out luciferase assays and RealTime PCR experiments and drafted the manuscript. AB carried out luciferase assays and RealTime PCR experiments. MV performed bioinformatics studies and sequence analysis and drafted the manuscript. CF and AR participated in co-immunoprecipitation assays and UV-cross linking experiments. AN participated in study design and revising the manuscript critically. PR participated in study design and coordination of the work and drafted the manuscript.
